# 2-Amino-4-methyl­pyridinium 2-carb­oxy­benzoate

**DOI:** 10.1107/S1600536810025900

**Published:** 2010-07-07

**Authors:** Ching Kheng Quah, Madhukar Hemamalini, Hoong-Kun Fun

**Affiliations:** a X-ray Crystallography Unit, School of Physics, Universiti Sains Malaysia, 11800 USM, Penang, Malaysia

## Abstract

In the title mol­ecular salt, C_6_H_9_N_2_^+^·C_8_H_5_O_4_^−^, the anion is stabilized by an intra­molecular O—H⋯O hydrogen bond, which generates an *S*(7) ring motif. In the crystal, the cations and anions are linked to form extended chains along [001] by O—H⋯O and N—H⋯O hydrogen bonds. Adjacent chains are crosslinked *via* C—H⋯O inter­actions into sheets lying parallel to (100).

## Related literature

For substituted pyridines, see: Pozharski *et al.* (1997 ▶); Katritzky *et al.* (1996 ▶). For details of hydrogen bonding, see: Scheiner (1997 ▶); Jeffrey & Saenger (1991 ▶); Jeffrey (1997 ▶). For hydrogen-bond motifs, see: Bernstein *et al.* (1995 ▶). For related structures, see: Quah *et al.* (2008*a* ▶,*b* ▶,*c* ▶). For reference bond lengths, see: Allen *et al.* (1987 ▶). For the stability of the temperature controller used for the data collection, see: Cosier & Glazer (1986 ▶).
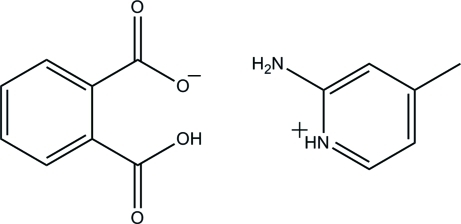


## Experimental

### 

#### Crystal data

C_6_H_9_N_2_^+^·C_8_H_5_O_4_^−^
*M* *_r_* = 274.27
Monoclinic, 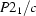

*a* = 13.0558 (15) Å
*b* = 6.9182 (8) Å
*c* = 14.2575 (17) Å
β = 90.218 (2)°
*V* = 1287.8 (3) Å^3^
*Z* = 4
Mo *K*α radiation
μ = 0.11 mm^−1^
*T* = 100 K
0.41 × 0.19 × 0.11 mm

#### Data collection

Bruker SMART APEXII DUO CCD diffractometer
Absorption correction: multi-scan (*SADABS*; Bruker, 2009 ▶) *T*_min_ = 0.958, *T*_max_ = 0.989
26319 measured reflections
3856 independent reflections
3332 reflections with *I* > 2σ(*I*)
*R*_int_ = 0.046

#### Refinement

*R*[*F* ^2^ > 2σ(*F* ^2^)] = 0.047
*wR*(*F* ^2^) = 0.131
*S* = 1.13
3856 reflections
233 parameters
H atoms treated by a mixture of independent and constrained refinement
Δρ_max_ = 0.41 e Å^−3^
Δρ_min_ = −0.33 e Å^−3^

### 

Data collection: *APEX2* (Bruker, 2009 ▶); cell refinement: *SAINT* (Bruker, 2009 ▶); data reduction: *SAINT*; program(s) used to solve structure: *SHELXTL* (Sheldrick, 2008 ▶); program(s) used to refine structure: *SHELXTL*; molecular graphics: *SHELXTL*; software used to prepare material for publication: *SHELXTL* and *PLATON* (Spek, 2009 ▶).

## Supplementary Material

Crystal structure: contains datablocks global, I. DOI: 10.1107/S1600536810025900/hb5537sup1.cif

Structure factors: contains datablocks I. DOI: 10.1107/S1600536810025900/hb5537Isup2.hkl

Additional supplementary materials:  crystallographic information; 3D view; checkCIF report

## Figures and Tables

**Table 1:** Hydrogen-bond geometry (Å, °)

*D*—H⋯*A*	*D*—H	H⋯*A*	*D*⋯*A*	*D*—H⋯*A*
O2—H1*O*2⋯O3	0.86	1.57	2.4009 (14)	163
N1—H1*N*1⋯O4^i^	0.94 (2)	1.77 (2)	2.6919 (16)	169 (2)
N2—H1*N*2⋯O3^i^	0.89 (2)	2.11 (2)	2.9881 (16)	166.6 (19)
N2—H2*N*2⋯O1^ii^	0.86 (2)	2.06 (2)	2.8888 (16)	164.5 (19)
C5—H5*A*⋯O2^iii^	0.953 (19)	2.532 (19)	3.4133 (17)	153.7 (16)

Symmetry codes: (i) 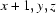; (ii) 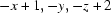; (iii) 
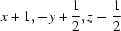
.

**Fig. 1.**
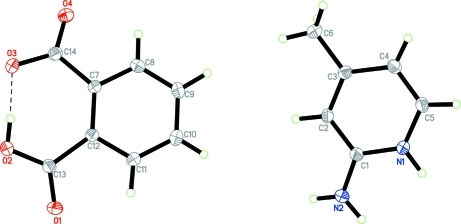
The molecular structure of (I), showing 50% probability displacement ellipsoids for non-H atoms. The intramolecular hydrogen bond is shown in dashed line.

**Fig. 2.**
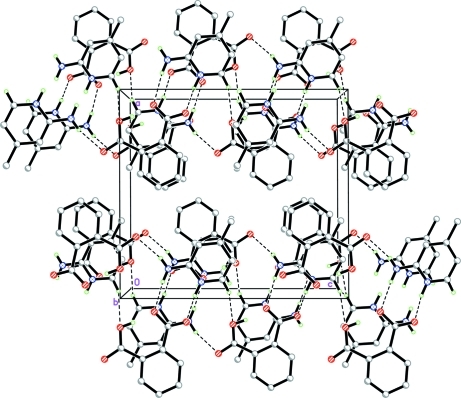
The crystal structure of (I) viewed along the b axis. H atoms not involved in intermolecular interactions (dashed lines) have been omitted for clarity.

## supplementary crystallographic information

### Comment

Pyridine and its derivatives play an important role in heterocyclic chemistry (Pozharski *et al.*, 1997; Katritzky *et al.*, 1996). They are often involved in hydrogen-bond interactions (Jeffrey & Saenger, 1991; Jeffrey, 1997; Scheiner, 1997). Since our aim is to study some interesting hydrogen-bonding interactions, the crystal structure of the title compound, (I), is presented here.

The asymmetric unit of the title compound contains one 2-amino-4-methylpyridinium cation and one 2-carboxybenzoate anion. A proton transfer from the carboxyl group of 2-carboxybenzoice acid to atom N1 of 2-amino-4-methylpyridinium resulted in the formation of ions. The bond lengths (Allen *et al.*, 1987) and angles in the title compound (Fig. 1) are within normal ranges and comparable with the related structures (Quah *et al.*, 2008*a*,*b*,*c*). The 2-amino-4-methylpyridinium cation is essentially planar, with the maximum deviation of 0.024 (1) Å for atom C2 and makes a dihedral angle of 19.56 (6)° with benzene (C7—C12) ring in 2-carboxybenzoate anion. The molecular structure is stabilized by an intramolecular O2—H1O2···O3 hydrogen bond which generates an *S*(7) ring motif (Bernstein *et al.*, 1995).

In the solid state, the cations and anions are linked to form extended chains along [0 0 1] by O–H···O and N–H···O hydrogen bonds (Table 1). The adjacent chains are cross-linked *via* C5–H5A···O2 interactions into two-dimensional networks (Fig. 2) parallel to the (1 0 0).

### Experimental

A hot methanol solution (20 ml) of 2-amino-4-methylpyridine (27 mg, Aldrich) and phthalic acid (41 mg, Merck) were mixed and warmed over a heating magnetic stirrer for a few minutes. The resulting solution was allowed to cool slowly at room temperature and colourless blocks of (I) appeared after a few days.

### Refinement

Atom H1O2 was located in a difference Fourier map and refined as riding with the parent atom with U_iso_(H) = 1.5U_eq_(O) [O2—H1O2 = 0.856 Å]. The remaining H atoms were located in a difference Fourier map and refined freely [N—H = 0.86 (2)–0.84 (2) Å and C—H = 0.892 (18)–1.00 (2) Å].

### Crystal data

**Table:**

C_6_H_9_N_2_^+^·C_8_H_5_O_4_^−^	*F*(000) = 576
*M**_r_* = 274.27	*D*_x_ = 1.415 Mg m^−^^3^
Monoclinic, *P*2_1_/*c*	Mo *K*α radiation, λ = 0.71073 Å
Hall symbol: -P 2ybc	Cell parameters from 6320 reflections
*a* = 13.0558 (15) Å	θ = 2.9–30.3°
*b* = 6.9182 (8) Å	µ = 0.11 mm^−^^1^
*c* = 14.2575 (17) Å	*T* = 100 K
β = 90.218 (2)°	Block, colourless
*V* = 1287.8 (3) Å^3^	0.41 × 0.19 × 0.11 mm
*Z* = 4	

### Data collection

**Table:**

Bruker SMART APEXII DUO CCD diffractometer	3856 independent reflections
Radiation source: fine-focus sealed tube	3332 reflections with *I* > 2σ(*I*)
graphite	*R*_int_ = 0.046
φ and ω scans	θ_max_ = 30.3°, θ_min_ = 2.9°
Absorption correction: multi-scan (*SADABS*; Bruker, 2009)	*h* = −18→18
*T*_min_ = 0.958, *T*_max_ = 0.989	*k* = −9→9
26319 measured reflections	*l* = −20→20

### Refinement

**Table:**

Refinement on *F*^2^	Primary atom site location: structure-invariant direct methods
Least-squares matrix: full	Secondary atom site location: difference Fourier map
*R*[*F*^2^ > 2σ(*F*^2^)] = 0.047	Hydrogen site location: inferred from neighbouring sites
*wR*(*F*^2^) = 0.131	H atoms treated by a mixture of independent and constrained refinement
*S* = 1.13	*w* = 1/[σ^2^(*F*_o_^2^) + (0.0537*P*)^2^ + 0.7248*P*] where *P* = (*F*_o_^2^ + 2*F*_c_^2^)/3
3856 reflections	(Δ/σ)_max_ = 0.001
233 parameters	Δρ_max_ = 0.41 e Å^−^^3^
0 restraints	Δρ_min_ = −0.33 e Å^−^^3^

### Special details

**Table:**

Experimental. The crystal was placed in the cold stream of an Oxford Cyrosystems Cobra open-flow nitrogen cryostat (Cosier & Glazer, 1986) operating at 100.0 (1) K.
Geometry. All e.s.d.'s (except the e.s.d. in the dihedral angle between two l.s. planes) are estimated using the full covariance matrix. The cell e.s.d.'s are taken into account individually in the estimation of e.s.d.'s in distances, angles and torsion angles; correlations between e.s.d.'s in cell parameters are only used when they are defined by crystal symmetry. An approximate (isotropic) treatment of cell e.s.d.'s is used for estimating e.s.d.'s involving l.s. planes.
Refinement. Refinement of *F*^2^ against ALL reflections. The weighted *R*-factor *wR* and goodness of fit *S* are based on *F*^2^, conventional *R*-factors *R* are based on *F*, with *F* set to zero for negative *F*^2^. The threshold expression of *F*^2^ > σ(*F*^2^) is used only for calculating *R*-factors(gt) *etc*. and is not relevant to the choice of reflections for refinement. *R*-factors based on *F*^2^ are statistically about twice as large as those based on *F*, and *R*- factors based on ALL data will be even larger.

### Fractional atomic coordinates and isotropic or equivalent isotropic displacement parameters (Å^2^)

**Table:**

	*x*	*y*	*z*	*U*_iso_*/*U*_eq_	
N1	0.92537 (9)	0.10419 (17)	0.64609 (8)	0.0185 (2)	
N2	0.85884 (10)	0.0110 (2)	0.78891 (8)	0.0230 (3)	
C1	0.84333 (10)	0.05716 (19)	0.69918 (9)	0.0174 (3)	
C2	0.74595 (10)	0.05677 (19)	0.65551 (9)	0.0173 (2)	
C3	0.73580 (10)	0.10080 (19)	0.56198 (9)	0.0173 (2)	
C4	0.82425 (10)	0.1503 (2)	0.50975 (9)	0.0188 (3)	
C5	0.91673 (10)	0.1521 (2)	0.55390 (9)	0.0190 (3)	
C6	0.63362 (11)	0.0948 (2)	0.51390 (11)	0.0233 (3)	
O1	0.29640 (8)	0.07772 (17)	1.07382 (7)	0.0256 (2)	
O2	0.14735 (7)	0.13709 (16)	1.00722 (7)	0.0220 (2)	
H1O2	0.1249	0.1484	0.9510	0.033*	
O3	0.07013 (7)	0.10883 (16)	0.85474 (7)	0.0232 (2)	
O4	0.11341 (8)	0.01778 (17)	0.71187 (7)	0.0255 (2)	
C7	0.25074 (10)	0.07219 (19)	0.81743 (9)	0.0169 (2)	
C8	0.31459 (11)	0.0630 (2)	0.73921 (10)	0.0252 (3)	
C9	0.42058 (12)	0.0674 (3)	0.74708 (11)	0.0343 (4)	
C10	0.46574 (12)	0.0806 (3)	0.83501 (11)	0.0307 (4)	
C11	0.40349 (10)	0.0909 (2)	0.91336 (10)	0.0212 (3)	
C12	0.29652 (10)	0.08811 (18)	0.90749 (9)	0.0158 (2)	
C13	0.24430 (10)	0.10053 (19)	1.00235 (9)	0.0178 (3)	
C14	0.13713 (10)	0.0649 (2)	0.79293 (10)	0.0186 (3)	
H2A	0.6866 (15)	0.030 (3)	0.6899 (13)	0.025 (5)*	
H4A	0.8212 (13)	0.180 (3)	0.4489 (13)	0.020 (4)*	
H5A	0.9801 (14)	0.181 (3)	0.5240 (13)	0.026 (5)*	
H6A	0.6231 (16)	0.198 (3)	0.4689 (15)	0.038 (6)*	
H6B	0.6280 (18)	−0.026 (4)	0.4780 (16)	0.044 (6)*	
H6C	0.5785 (19)	0.100 (3)	0.5570 (18)	0.050 (7)*	
H8A	0.2830 (17)	0.056 (3)	0.6805 (16)	0.038 (6)*	
H9A	0.4616 (17)	0.062 (3)	0.6884 (16)	0.039 (6)*	
H10A	0.5374 (18)	0.080 (3)	0.8406 (15)	0.037 (6)*	
H11A	0.4322 (14)	0.106 (2)	0.9756 (13)	0.018 (4)*	
H1N1	0.9896 (18)	0.087 (3)	0.6745 (15)	0.038 (6)*	
H1N2	0.9214 (18)	0.022 (3)	0.8138 (15)	0.037 (6)*	
H2N2	0.8067 (17)	−0.025 (3)	0.8207 (14)	0.028 (5)*	

### Atomic displacement parameters (Å^2^)

**Table:**

	*U*^11^	*U*^22^	*U*^33^	*U*^12^	*U*^13^	*U*^23^
N1	0.0148 (5)	0.0233 (6)	0.0174 (5)	−0.0020 (4)	−0.0001 (4)	0.0000 (4)
N2	0.0200 (6)	0.0340 (7)	0.0150 (5)	−0.0024 (5)	−0.0008 (4)	0.0032 (5)
C1	0.0176 (6)	0.0188 (6)	0.0159 (6)	−0.0008 (4)	0.0006 (4)	−0.0010 (4)
C2	0.0148 (5)	0.0198 (6)	0.0172 (6)	−0.0013 (4)	0.0019 (4)	−0.0013 (5)
C3	0.0169 (6)	0.0176 (6)	0.0175 (6)	0.0003 (4)	−0.0015 (4)	−0.0015 (4)
C4	0.0204 (6)	0.0204 (6)	0.0156 (6)	−0.0002 (5)	0.0010 (5)	0.0009 (5)
C5	0.0184 (6)	0.0208 (6)	0.0178 (6)	−0.0020 (5)	0.0031 (5)	0.0009 (5)
C6	0.0176 (6)	0.0309 (7)	0.0212 (6)	−0.0006 (5)	−0.0036 (5)	0.0001 (6)
O1	0.0203 (5)	0.0412 (6)	0.0153 (4)	−0.0024 (4)	−0.0015 (4)	−0.0009 (4)
O2	0.0181 (5)	0.0317 (5)	0.0163 (4)	0.0040 (4)	0.0016 (3)	−0.0018 (4)
O3	0.0147 (4)	0.0350 (6)	0.0199 (5)	0.0033 (4)	−0.0001 (4)	0.0006 (4)
O4	0.0199 (5)	0.0366 (6)	0.0201 (5)	0.0000 (4)	−0.0039 (4)	−0.0031 (4)
C7	0.0149 (5)	0.0197 (6)	0.0160 (5)	0.0004 (4)	−0.0009 (4)	0.0009 (4)
C8	0.0204 (6)	0.0397 (8)	0.0156 (6)	0.0001 (6)	0.0003 (5)	−0.0003 (6)
C9	0.0196 (7)	0.0636 (12)	0.0197 (7)	−0.0003 (7)	0.0047 (5)	−0.0031 (7)
C10	0.0152 (6)	0.0530 (10)	0.0239 (7)	0.0010 (6)	0.0017 (5)	−0.0021 (7)
C11	0.0164 (6)	0.0292 (7)	0.0181 (6)	0.0014 (5)	−0.0014 (5)	−0.0003 (5)
C12	0.0155 (5)	0.0170 (6)	0.0150 (5)	0.0004 (4)	0.0003 (4)	0.0006 (4)
C13	0.0172 (6)	0.0199 (6)	0.0163 (6)	−0.0019 (5)	0.0010 (4)	−0.0013 (5)
C14	0.0169 (6)	0.0202 (6)	0.0188 (6)	−0.0004 (5)	−0.0012 (5)	0.0026 (5)

### Geometric parameters (Å, °)

**Table:**

N1—C1	1.3535 (17)	O1—C13	1.2331 (16)
N1—C5	1.3599 (17)	O2—C13	1.2929 (16)
N1—H1N1	0.94 (2)	O2—H1O2	0.8555
N2—C1	1.3332 (17)	O3—C14	1.2807 (17)
N2—H1N2	0.89 (2)	O4—C14	1.2391 (17)
N2—H2N2	0.86 (2)	C7—C8	1.3963 (19)
C1—C2	1.4136 (18)	C7—C12	1.4186 (17)
C2—C3	1.3738 (18)	C7—C14	1.5233 (18)
C2—H2A	0.94 (2)	C8—C9	1.388 (2)
C3—C4	1.4183 (19)	C8—H8A	0.93 (2)
C3—C6	1.4981 (18)	C9—C10	1.386 (2)
C4—C5	1.3596 (19)	C9—H9A	1.00 (2)
C4—H4A	0.892 (18)	C10—C11	1.386 (2)
C5—H5A	0.953 (19)	C10—H10A	0.94 (2)
C6—H6A	0.97 (2)	C11—C12	1.3988 (18)
C6—H6B	0.98 (2)	C11—H11A	0.967 (18)
C6—H6C	0.95 (3)	C12—C13	1.5195 (18)
			
C1—N1—C5	122.43 (12)	H6B—C6—H6C	108 (2)
C1—N1—H1N1	115.9 (14)	C13—O2—H1O2	107.5
C5—N1—H1N1	121.3 (14)	C8—C7—C12	118.39 (12)
C1—N2—H1N2	119.7 (14)	C8—C7—C14	113.53 (12)
C1—N2—H2N2	117.3 (13)	C12—C7—C14	128.08 (12)
H1N2—N2—H2N2	122.9 (19)	C9—C8—C7	122.17 (13)
N2—C1—N1	118.46 (12)	C9—C8—H8A	120.7 (14)
N2—C1—C2	123.73 (13)	C7—C8—H8A	117.1 (14)
N1—C1—C2	117.80 (12)	C10—C9—C8	119.66 (14)
C3—C2—C1	120.69 (12)	C10—C9—H9A	122.3 (13)
C3—C2—H2A	118.3 (12)	C8—C9—H9A	118.0 (13)
C1—C2—H2A	121.0 (12)	C11—C10—C9	118.92 (14)
C2—C3—C4	119.17 (12)	C11—C10—H10A	121.3 (13)
C2—C3—C6	121.37 (12)	C9—C10—H10A	119.8 (13)
C4—C3—C6	119.46 (12)	C10—C11—C12	122.65 (13)
C5—C4—C3	118.86 (12)	C10—C11—H11A	121.2 (11)
C5—C4—H4A	119.0 (11)	C12—C11—H11A	116.1 (11)
C3—C4—H4A	122.2 (11)	C11—C12—C7	118.20 (12)
C4—C5—N1	121.03 (12)	C11—C12—C13	113.40 (11)
C4—C5—H5A	124.5 (11)	C7—C12—C13	128.40 (12)
N1—C5—H5A	114.4 (11)	O1—C13—O2	121.18 (12)
C3—C6—H6A	114.0 (12)	O1—C13—C12	118.71 (12)
C3—C6—H6B	109.1 (14)	O2—C13—C12	120.10 (12)
H6A—C6—H6B	105.8 (19)	O4—C14—O3	122.37 (12)
C3—C6—H6C	112.3 (15)	O4—C14—C7	117.53 (12)
H6A—C6—H6C	107.2 (19)	O3—C14—C7	120.09 (12)
			
C5—N1—C1—N2	179.65 (13)	C10—C11—C12—C7	0.6 (2)
C5—N1—C1—C2	0.7 (2)	C10—C11—C12—C13	179.86 (15)
N2—C1—C2—C3	−178.20 (13)	C8—C7—C12—C11	−0.9 (2)
N1—C1—C2—C3	0.7 (2)	C14—C7—C12—C11	179.59 (13)
C1—C2—C3—C4	−1.1 (2)	C8—C7—C12—C13	179.96 (13)
C1—C2—C3—C6	178.00 (13)	C14—C7—C12—C13	0.5 (2)
C2—C3—C4—C5	0.2 (2)	C11—C12—C13—O1	−12.47 (18)
C6—C3—C4—C5	−178.95 (13)	C7—C12—C13—O1	166.68 (13)
C3—C4—C5—N1	1.2 (2)	C11—C12—C13—O2	166.57 (13)
C1—N1—C5—C4	−1.6 (2)	C7—C12—C13—O2	−14.3 (2)
C12—C7—C8—C9	0.5 (2)	C8—C7—C14—O4	12.57 (19)
C14—C7—C8—C9	−179.94 (16)	C12—C7—C14—O4	−167.93 (14)
C7—C8—C9—C10	0.3 (3)	C8—C7—C14—O3	−166.62 (14)
C8—C9—C10—C11	−0.6 (3)	C12—C7—C14—O3	12.9 (2)
C9—C10—C11—C12	0.2 (3)		

### Hydrogen-bond geometry (Å, °)

**Table:**

*D*—H···*A*	*D*—H	H···*A*	*D*···*A*	*D*—H···*A*
O2—H1O2···O3	0.86	1.57	2.4009 (14)	163
N1—H1N1···O4^i^	0.94 (2)	1.77 (2)	2.6919 (16)	169 (2)
N2—H1N2···O3^i^	0.89 (2)	2.11 (2)	2.9881 (16)	166.6 (19)
N2—H2N2···O1^ii^	0.86 (2)	2.06 (2)	2.8888 (16)	164.5 (19)
C5—H5A···O2^iii^	0.953 (19)	2.532 (19)	3.4133 (17)	153.7 (16)

Symmetry codes: (i) *x*+1, *y*, *z*; (ii) −*x*+1, −*y*, −*z*+2; (iii) *x*+1, −*y*+1/2, *z*−1/2.
